# Evaluation of a New Recombinant Oncolytic Vaccinia Virus Strain GLV-5b451 for Feline Mammary Carcinoma Therapy

**DOI:** 10.1371/journal.pone.0104337

**Published:** 2014-08-05

**Authors:** Marion Adelfinger, Ivaylo Gentschev, Julio Grimm de Guibert, Stephanie Weibel, Johanna Langbein-Laugwitz, Barbara Härtl, Hugo Murua Escobar, Ingo Nolte, Nanhai G. Chen, Richard J. Aguilar, Yong A. Yu, Qian Zhang, Alexa Frentzen, Aladar A. Szalay

**Affiliations:** 1 Department of Biochemistry, University of Wuerzburg, Wuerzburg, Germany; 2 Genelux Corporation, San Diego Science Center, San Diego, California, United States of America; 3 Genelux GmbH, Bernried, Germany; 4 Small Animal Clinic, University of Veterinary Medicine, Hannover, Germany; 5 Division of Medicine Clinic III, Hematology, Oncology and Palliative Medicine University of Rostock, Rostock, Germany; 6 Department of Radiation Medicine and Applied Sciences, Rebecca & John Moores Comprehensive Cancer Center, University of California San Diego, La Jolla, California, United States of America; 7 Rudolf Virchow Center for Experimental Biomedicine, University of Wuerzburg, Wuerzburg, Germany; 8 Institute for Molecular Infection Biology, University of Wuerzburg, Wuerzburg, Germany; McMaster University, Canada

## Abstract

Virotherapy on the basis of oncolytic vaccinia virus (VACV) infection is a promising approach for cancer therapy. In this study we describe the establishment of a new preclinical model of feline mammary carcinoma (FMC) using a recently established cancer cell line, DT09/06. In addition, we evaluated a recombinant vaccinia virus strain, GLV-5b451, expressing the anti-vascular endothelial growth factor (VEGF) single-chain antibody (scAb) GLAF-2 as an oncolytic agent against FMC. Cell culture data demonstrate that GLV-5b451 virus efficiently infected, replicated in and destroyed DT09/06 cancer cells. In the selected xenografts of FMC, a single systemic administration of GLV-5b451 led to significant inhibition of tumor growth in comparison to untreated tumor-bearing mice. Furthermore, tumor-specific virus infection led to overproduction of functional scAb GLAF-2, which caused drastic reduction of intratumoral VEGF levels and inhibition of angiogenesis.

In summary, here we have shown, for the first time, that the vaccinia virus strains and especially GLV-5b451 have great potential for effective treatment of FMC in animal model.

## Introduction

Mammary gland tumors are among the most frequently observed tumors in older female cats [1], [2]. In contrast to dogs and humans, between 85% and 93% of feline mammary tumors are malignant [1], [3]. The prognosis of feline patients with advanced mammary malignancy is poor, because this disease is also very often associated with formation of metastases in one or more organs [1], [4]–[6]. Despite progress in the diagnosis and treatment of advanced feline cancer, overall patient treatment outcome has not been substantially improved in the past. Therefore, there is an urgent need to identify novel agents for therapy of advanced feline cancer. One of the most promising novel cancer therapies is oncolytic virotherapy. This method is based on the capacity of oncolytic viruses to preferentially infect and lyse cancer cells without causing excessive damage to surrounding normal tissue. Several oncolytic viruses have been successfully tested for human therapy in preclinical and clinical settings (for a review see [7]). However, in contrast to human studies, only one clinical trial with moderate success for feline cancer patients has been reported [8].

In the present study, we evaluated for the first time the therapeutic potential of the new recombinant oncolytic vaccinia virus GLV-5b451 expressing the anti-VEGF single-chain antibody (scAb) GLAF-2 against feline mammary carcinoma (FMC). GLV-5b451 was derived from the oncolytic vaccinia virus LIVP 6.1.1 [9] by inserting the *glaf-2* gene [10] encoding the GLAF-2 antibody under the control of the vaccinia virus synthetic early-late (SEL) promoter [11] into the *J2R* (encoding thymidine kinase) locus. VEGF or VEGF-A is a potent key regulator of tumor angiogenesis and several anti-VEGF strategies have been developed for the treatment of different cancer patients [12]–[15]. It was shown that overexpression of VEGF in malignant tissues does correlate very well with an unfavorable prognosis for feline cancer patients with FMC [16], [17]. Therefore, new methods or vectors allowing more specific targeting and inactivation of VEGF inside of tumor tissues are urgently needed. We have already shown that VACV expressing anti-VEGF antibodies exhibited significant reduction of tumor growth and enhanced inhibition of angiogenesis in comparison to control animals [10], [11], [18], [19].

Here, we report that the virus-mediated oncolytic and immunological effects upon colonization of tumors with GLV-5b451 followed by constitutive intratumoral production of functional scAb GLAF-2 led to significant inhibition of tumor growth and tumor angiogenesis in mice with feline mammary tumor xenografts.

## Materials and Methods

### Ethics statement

All mice animal experiments were carried out in accordance with protocols approved by the Institutional Animal Care and Use Committee (IACUC) of Explora Biolabs (San Diego, CA, USA; protocol number: EB11-025) and/or the government of Unterfranken, Germany, according to the German Animal Welfare Act (TierSchG)“ (permit number: 55.2-2531.01-17/08 and 55.2-2531.01-24/12).

The feline cell line DT09/06 was derived from a mammary carcinoma (Small Animal Clinic, University of Veterinary Medicine, Hannover, Germany). Preservation of diagnostic samples taken from animals as part of a medical procedure is a routine practice and does not require approval by a review board or consent of the owner. There was no need for study approval by a named review board institution or ethics committee because the mammary tumor was removed as part of the medical care of the patient. Once a tumor is removed from a patient, the tissue becomes a diagnostic sample. Isolation of a small section of the tumor does not interfere with diagnostic analysis of the tumor. Written consent of the owner was obtained for tumor removal and diagnostic procedures. This consent form is part of the patient's medical record.

The F1B cell line was derived from the swollen left submandibular lymph node of a 3-year-old female cat with clinically diagnosed lymphoma [20].

The canine cell line MTH52c was derived from a malignant small-cell carcinoma and was already described by Sterenczak *et al.* and Gentschev *et al.* [21], [22].

### Donor

The cell line DT09/06 was derived from a tumor of a thirteen year old, female, unspayed British Shorthair cat (Small Animal Clinic, University of Veterinary Medicine, Hannover, Germany). Histopathology revealed an ulcerating stage IV mammary carcinoma with infiltrative growth and planar necrosis, fused with the abdominal wall. Tumor cell emboli were detected in the lymphatic vessels while the performed blood count displayed hyperproteinaemia and leukocytosis. Prognosis was considered unfavorable due to high risk of relapse and metastasis. The patient was prepared for palliative operation by combined administration of antibiotic amoxicillin/clavulanic acid and the non-steroidal anti-inflammatory drug meloxicam, resulting in a declining inflammation of the mamma. Subsequently, surgical treatment was performed, removing part of the inguinal region including the fascia and the mammary tumor itself. The feline patient was euthanized three weeks after the operation due to dyspnea.

### Cell culture

African green monkey kidney fibroblasts (CV-1) and feline lymphoma F1B cells (CRL-6168) were obtained from the American Type Culture Collection (ATCC). DT09/06 cells were derived from a feline patient with mammary carcinoma (this study) and the canine cell line MTH52c was derived from a malignant small-cell carcinoma [21], [22].

Cells were cultured in DMEM supplemented with antibiotic-solution (100 U/ml penicillin G, 100 units/ml streptomycin) and 10% fetal bovine serum (FBS; PAA Laboratories, Pasching, Austria) for CV-1 and F1B and 20% FBS for MTH52c. DT09/06 cells were cultured in minimum essential medium with Earle's salts (MEM) supplemented with 2 mM glutamine, 100 U/mL penicillin G, 100 µg/mL streptomycin, 1 mM sodium pyruvate, 0.1 mM nonessential amino acids (MEM-C), and 10% FBS. All cell lines were cultured at 37°C and 5% CO_2_ in a humidified incubator.

### ELISA

For the quantitative determination of feline VEGF concentrations in DT09/06 cell culture supernatants, 5×10^6^ cells were cultured in MEM containing 10% FBS. Cell culture supernatants were collected at 24, 48 and 72 h and stored at −20°C.

Lysates of virus-treated and untreated DT09/06 primary tumors were utilized for determination of VEGF presence in the tumor tissue.

Concentrations of VEGF were determined by VEGF ELISA kit (Thermo Scientific, Rockford, USA) developed for detection of human VEGF (cross-reacts approximately 82% to recombinant feline VEGF; R&D Systems, Inc., catalog number DVE00, page 11,www.RnDSystem.com), in accordance with the manufacturer's directions.

For the determination of the affinity and cross-reactivity of GLAF-2 to VEGF from different species, an ELISA was performed. For this purpose recombinant feline (5844-CV-010, R&D Systems, Minneapolis, MN, USA), murine (V4512, Sigma-Aldrich, St. Louis, MO, USA) and human (V7259, Sigma-Aldrich) VEGF proteins were pre-coated at a concentration of 100 ng/well in 96-well ELISA plates and incubated overnight at 4°C. The wells were washed once with bidest. water and twice with PBS/0.05% Tween (PBST) and blocked with 100 µl 1% w/v Blocker Casein in PBS (Pierce, 37528) for 2 h at 37°C. After washing the wells four times with PBST, wells were incubated with seven two-fold dilution series of GLAF-2 (2000 to 31.25 ng/ml, GenScript, *E.coli* expressed and purified tag-free) for 1 h at room temperature (RT). PBS was used as a negative control. The wells were washed again and incubated with a polyclonal rabbit anti-GLAF-2 antibody (1∶1000, GenScript) for 1 h at RT. After washing the wells were incubated with a HRP-conjugated polyclonal goat anti-rabbit IgG (H+L) (170-6515, Biorad, 1∶5000) for 1 h at RT. The plate was washed and staining was developed using TMB (T0440, Sigma-Aldrich) and stopped with 2 N HCl. The ELISA was read at a wavelength of 450 nm.

### Virus strains

Vaccinia virus strain LIVP 6.1.1 was derived from LIVP (Lister strain, Institute of Viral Preparations, Moscow, Russia) and is an oncolytic virus strain designed to locate, enter, colonize and destroy cancer cells without harming healthy tissues or organs [9].

GLV-5b451 virus was derived from the oncolytic vaccinia virus LIVP 6.1.1 by inserting the *glaf-2* gene [10] encoding the GLAF-2 antibody under the control of the vaccinia virus synthetic early-late (SEL) promoter [11] into the *J2R* (encoding thymidine kinase) locus (this study).

### Cell viability assays

DT09/06 or MTH52c cells were seeded in 24-well plates (Greiner Bio-One, Frickenhausen, Germany). After 24 h in culture, DT09/06 or MTH52c cells were infected with either LIVP 6.1.1 or GLV-5b451 using multiplicities of infection (MOI) of 0.1 and 1.0. The cells were incubated at 37°C for 1 h, then the infection medium was removed and subsequently the cells were incubated in fresh growth medium. The amount of viable cells after infection was measured using 3-(4,5-dimethylthiazol-2-yl)-2,5-diphenyltetrazolium bromide (MTT) (Sigma, Taufkirchen, Germany). At 24, 48, 72, or 96 h post infection of cells, the medium was replaced by 0.5 ml MTT solution at a concentration of 2.5 mg/ml MTT dissolved in DMEM without phenol red and incubated for 2 h at 37°C in a 5% CO_2_ atmosphere. After removal of the MTT solution, the color reaction was stopped by adding 1 N HCl diluted in isopropanol. The optical density was then measured at a wavelength of 570 nm in a Tecan Sunrise Remote microplate reader (Tecan, Männedorf, Switzerland). Uninfected cells were used as reference and were considered as 100% viable.

### Viral replication

For the viral replication assay, DT09/06 cancer cells were infected with LIVP 6.1.1 or GLV-5b451 at an MOI of 0.1. After one hour of incubation at 37°C, the infection medium was removed and replaced by fresh growth medium. After 1, 24, 48, 72 and 96 hours, the cells and supernatants were harvested. Following three freeze-thaw cycles and three times sonification (3×30 s), serial dilutions of the supernatants and lysates were titered by standard plaque assay on CV-1 cells. All samples were measured in triplicate.

### Western blot analysis

For detection of virus encoded proteins, DT09/06 cells were harvested and resuspended in SDS sample buffer at 24, 48, 72, or 96 hours post virus infection (hpvi). DT09/06 tumors were harvested 28 days post infection (dpi) and shredded in SDS sample buffer using shredder tubes (Peqlab, Erlangen, Germany). Samples were separated by 10% SDS-Polyacrylamide gel electrophoresis and subsequently transferred onto a nitrocellulose membrane (Whatman GmbH, Dassel, Germany). After blocking in 5% skim milk in PBS, the membrane was incubated with rabbit anti-G6 antibody (affinity-purified polyclonal antibody raised against a GLAF-2 peptide, GenScript, Piscataway, NJ, USA) for detection of scAb GLAF-2 or polyclonal rabbit anti-vaccinia virus antibody (ab35219 Abcam, Cambridge, UK). The primary antibodies were detected using horseradish peroxidase-conjugated anti-rabbit (ab6721, Abcam, Cambridge, UK) secondary antibody, followed by enhanced chemiluminescence detection.

### Vaccinia virus-mediated therapy of DT09/06 xenografts

Tumors were generated by implanting 1×10^7^ feline mammary carcinoma DT09/06 cells subcutaneously into the right hind leg of 6- to 8-week-old female nude mice [Hsd:Athymic Nude-*Foxn1* ^nu^; Harlan, Netherlands]. Tumor growth was monitored twice weekly in two dimensions using a digital caliper. Tumor volume was calculated as [(length×width^2^)/2]. On day 28, when tumor volume reached approximately 400 mm^3^, groups of mice (n = 13) were injected intravenously (i.v.) either with 1×10^7^ pfu of GLV-5b451 or LIVP 6.1.1 virus or PBS (control) into the tail vein. The significance of the results was calculated by two-way analysis of variance (ANOVA) with Bonferroni post-test. Results are displayed as means +/− standard deviation (SD). P values of <0.05 were considered significant. Mice were monitored for change in body weight and signs of toxicity.

### Histological and PCR genetic analysis of tumors

The spontaneous feline tumor surgically removed from a patient at the University of Hannover, Germany was used for histopathologic analysis. Portions of the tissues were paraffin-embedded, sectioned and stained with hematoxylin and eosin (H&E) to identify the tumor type. The feline mammary tumor was classified histologically as carcinoma.

Tumor cell lines or xenograft tissue samples were analyzed for the presence of DT09/06 cells by duplex PCR. For this purpose, DT09/06 cells or DT09/06 primary tumors were processed by DNeasy Blood & Tissue Kit (50) (Qiagen, Hilden Germany) to isolate total DNA and then analyzed by PCR, using DNA Polymerase (Phusion, Finnzymes, Espoo, Finland) and the primers for feline 12S rRNA gene (for the identification of DT09/06 cells, forward: 5′-AATTGAATCGGGCCATGAA-3′ and reverse: 5′-CGACTTATCTCCTCTTGTGGGTGT-3′); for murine 12S rRNA gene (as control, forward: 5′-AAATCCAACTTATATGTGAAAATTCATTGT-3′ and reverse: 5′-TGGGTCTTTAGCTATCGTCGATCAT-3′) [23]. The PCR reaction was run in a T-Gradient Thermoblock PCR machine (Biometra, Göttingen, Germany) under the following cycling conditions: initial heat-denaturation step at 93°C for 2 min, followed by 35 cycles of 93°C/30 sec, 60°C (cat) or 55°C (mouse)/30 sec, and 72°C/45 sec. The last extension step at 72°C was maintained for 3 min. The designed primers generated specific fragments of 108 or 96 bp in length for cat or mouse tissues, respectively.

For histological studies of the xenograft model, tumors were excised and snap-frozen in liquid N_2_, followed by fixation in 4% paraformaldehyde/PBS at pH 7.4 for 16 h at 4°C. After dehydration in 10% and 30% sucrose (Carl Roth, Karlsruhe, Germany) specimens were embedded in Tissue-Tek O.C.T. (Sakura Finetek Europe B.V., Alphen aan den Rijn, Netherlands). Tissue samples were sectioned (10 µm thickness) with the cryostat 2800 Frigocut (Leica Microsystems GmbH, Wetzlar, Germany).

A part of tissue sectioning was performed as described [24], [25]. In this case, VACVs were labeled using polyclonal rabbit anti-vaccinia virus (anti-VACV) antibody (Abcam, Cambridge, UK), which was stained using Cy2-conjugated donkey anti-rabbit secondary antibodies obtained from dianova (Hamburg, Germany). Endothelial blood vessel cells were stained with a monoclonal rat anti-mouse CD31 antibody (BD Biosciences, Franklin Lakes, NJ, USA) and a Cy3-conjugated donkey anti-rat secondary antibody from dianova.

Immune cells were labeled using rat anti-mouse MHCII antibody detecting a polymorphic determinant present on B cells, monocytes, macrophages and dendritic cells (eBioscience, San Diego, CA, USA) and Cy3-conjugated secondary antibody (donkey) obtained from dianova.

The fluorescence-labeled preparations were examined using a TCS SP2 AOBS confocal laser microscope (Leica Microsystems GmbH, Wetzlar, Germany) equipped with the LCS 2.16 software (1024×1024 pixel RGB-color images) and an Axiovert 200 M microscope (Carl Zeiss Microscopy GmbH, Göttingen, Germany) with Axiovision 4.5 software (1388×1040 pixel gray scale images). Digital images were processed with Photoshop 7.0 (Adobe Systems, Mountain View, CA, USA).

### Measurement of blood vessel density in the tumor tissues

Blood vessel density was measured in digital images (×100 magnification) of CD31-labelled 10-µm-thick tumor cross-sections using Leica LCS 2.16 software. Eight images per tumor were analyzed per staining (3 tumors per group, 2 sections of each tumor and 8 images per section). Tonal correction for all images was identical to ensure clear visibility of all detectable blood vessels and comparability of the results. All blood vessels were counted to obtain the vessel density per image.

## Results

### The newly established feline mammary cell line DT09/06 is tumorigenic in female nude mice

In cell culture, the newly isolated feline mammary DT09/06 cells were spindle-shaped with long extensions and did not form closed monolayers (**Figure S1A**). The doubling time of these cells was 22.46 h under these cell culture conditions (**Figure S1B**). The tumorigenic potential of DT09/06 was examined in 6- to 8-week-old female nude mice. For this purpose, four different doses of 5×10^5^, 1×10^6^, 5×10^6^ or 1×10^7^ DT09/06 cells were subcutaneously implanted into the right hind leg of the animals. Ninety-six percent of the DT09/06-implanted mice developed a detectable tumor mass. Eight to nine weeks post implantation about 56% of mice developed tumors of 2000 to 3000 mm^3^. The histological examination of the primary tumors revealed atypical epithelial cells arranged in neoplastic emboli bundles and streams (**Figure S1C**).

Finally, the feline origin of DT09/06 cells or DT09/06 primary tumors was confirmed through duplex PCR analysis (**Figure S1D**). The data demonstrated that the feline DT09/06 cell line was tumorigenic in female nude mice.

### Expression of VEGF protein in DT09/06 cancer cells under cell culture conditions

**Figure 1:**
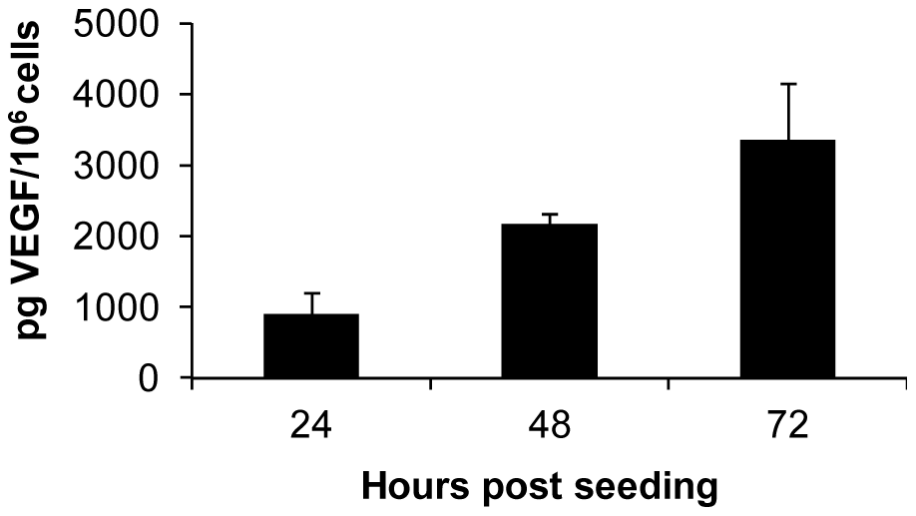
VEGF expression in feline mammary cancer DT09/06 cells under cell culture conditions. DT09/06 cells in culture conditions were washed with PBS and cultured in fresh medium with 2% FCS. Culture supernatants were harvested at 24, 48 and 72 h. Concentration of VEGF in supernatants was represented as pg/10^6^ cells. Each value represents the mean (n = 3) +/− standard deviations (SD).

VEGF is a potent mediator of both angiogenesis and vasculogenesis in cats and has been proposed as a prognostic indicator in invasive feline mammary carcinomas (FMCs) [16] [26]–[28]. Therefore, we first analyzed the VEGF expression of the feline mammary cancer cell line DT09/06 under cell culture conditions (**Figure 1**). VEGF concentrations were determined using a VEGF ELISA kit (Thermo Scientific, Rockford, USA) developed for detection of human VEGF, in accordance with the manufacturer's directions. VEGF levels in the supernatant of DT09/06 cells were 905.6±296.47 pg/10^6^ cells (24 hours), 2171.95±149.15 pg/10^6^ cells (48 hours) and 3354.95±798.93 pg/10^6^ cells (72 hours). The data revealed that DT09/06 cells constitutively produced VEGF at all tested time points.

### Oncolytic vaccinia virus GLV-5b451 expressing the anti-vascular endothelial growth factor (VEGF) single-chain antibody (scAb) GLAF-2 efficiently kills feline mammary carcinoma DT09/06 cells

**Figure 2:**
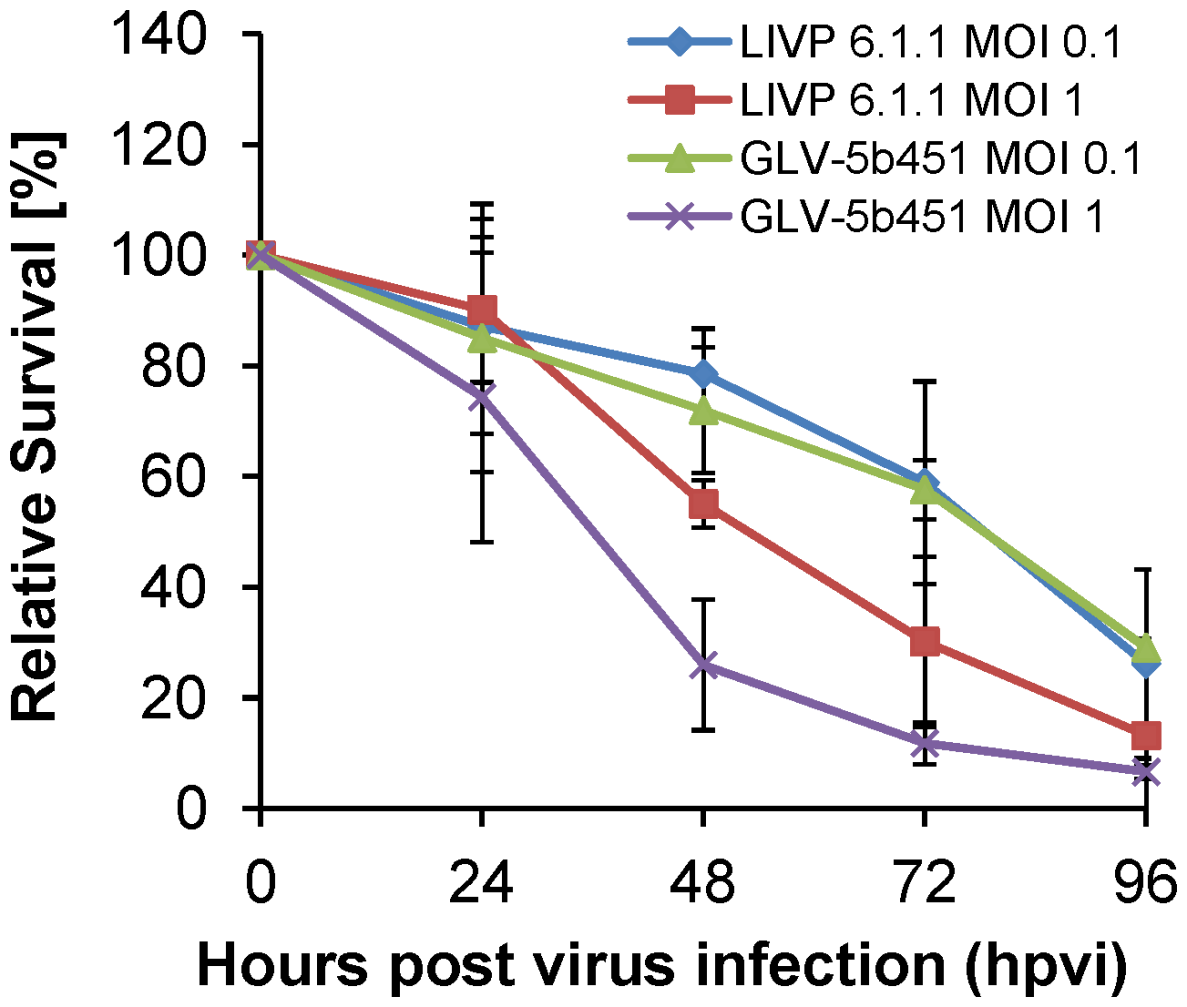
Viability of the feline mammary carcinoma DT09/06 cells after LIVP 6.1.1 or GLV-5b451 infection at MOIs of 0.1 and 1.0. Viable cells were detected using 3-(4,5-dimethylthiazol-2-yl)-2,5-diphenyltetrazolium bromide (MTT). Mean values (n = 3) and standard deviations are shown as percentages of respective controls. The data represent two independent experiments. There were no significant differences between groups (P>0.05).

Five ×10^4^ DT09/06 cells/well were seeded one day prior to infection in 24-well plates and were then infected with either GLV-5b451 or LIVP 6.1.1 (non-GLAF-2 expressing parental virus strain) at MOIs of 1.0 and 0.1, respectively. Cell viability was analyzed at 24, 48, 72 and 96 hours post virus infection (hpvi) by MTT-assays (**Figure 2**). Ninety-six hours after GLV-5b451 infection at MOIs of 0.1 and 1.0, only 29.07% and 6.62% DT09/06 cells survived the treatment, respectively. At the same time point and MOIs, we found 26.15% and 13.17% viable DT09/06 cells after LIVP 6.1.1 infection.

The data demonstrated that GLV-5b451 and LIVP 6.1.1 efficiently infected and destroyed feline mammary carcinoma DT09/06 cells under these cell culture conditions. There was no statistically significant difference in the number of viable cells between the two virus strains. In addition, similar oncolytic effect was found after GLV-5b451 infection of the feline lymphoma F1B cells and canine mammary carcinoma MTH52c cells (**Figure S2**).

### GLV-5b451 efficiently replicates in feline mammary carcinoma DT09/06 cells

**Figure 3:**
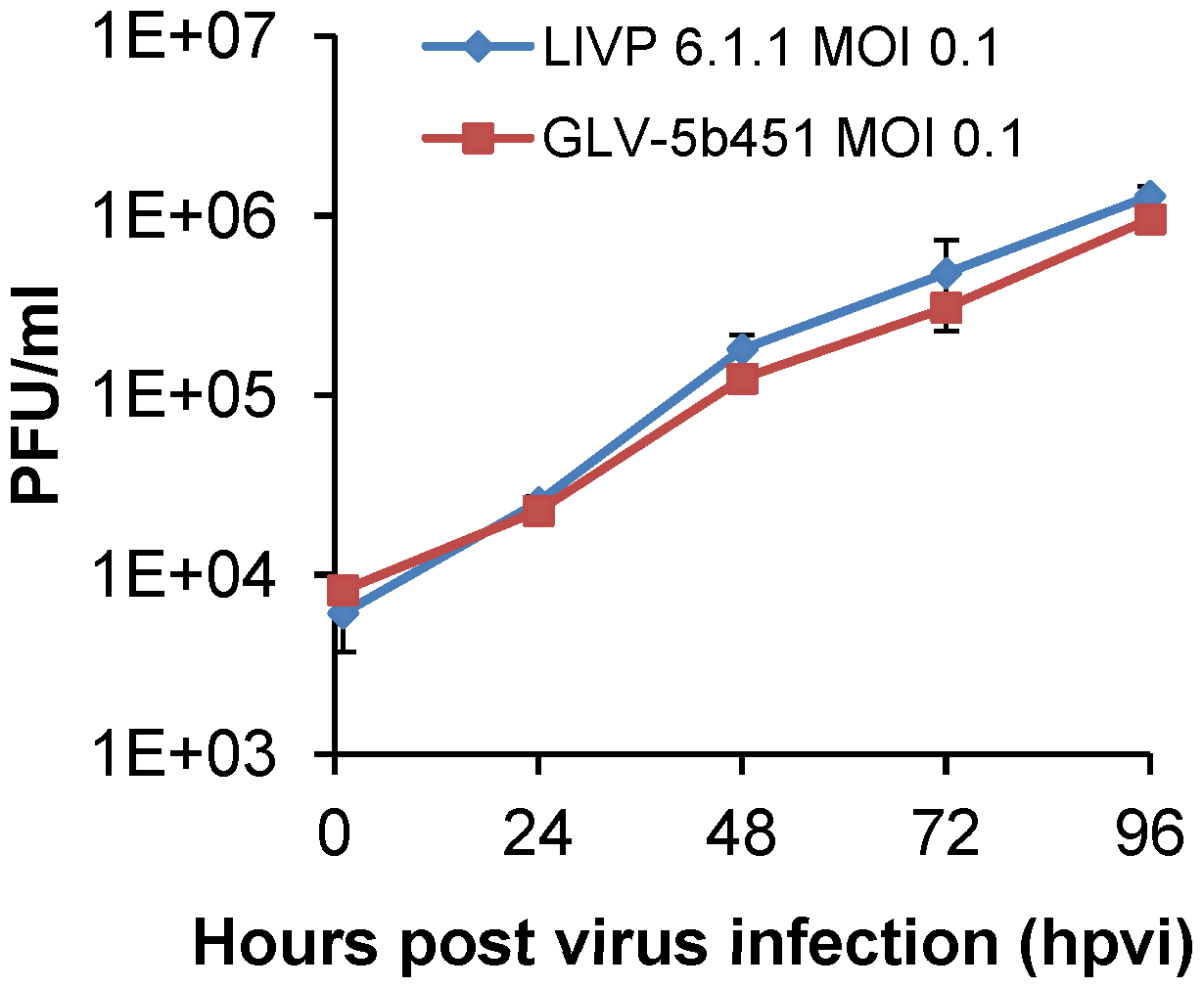
Comparison of the replication capacity of the vaccinia virus strains LIVP 6.1.1 or GLV-5b451 in DT09/06 cells. For the viral replication assay, DT09/06 cells grown in 24-well plates were infected with either LIVP 6.1.1 or GLV-5b451 at an MOI of 0.1. Cells and supernatants were collected for the determination of virus titer at various time points. Viral titers were determined as pfu per well in triplicates by standard plaque assay in CV-1 cell monolayers. Averages plus standard deviation are plotted. The data represent two independent experiments.

DT09/06 cells were infected with either GLV-5b451 or LIVP 6.1.1 at an MOI of 0.1, in order to test the ability of GLV-5b451 to infect and efficiently replicate in feline mammary carcinoma cells. Standard plaque assays were performed for all samples to determine the viral titers at different time points during the course of infection (**Figure 3**). The maximum viral titers (total) were observed at 96 hours post virus infection (hpvi) for both GLV-5b451 (2.98×10^6^ pfu/ml) and LIVP 6.1.1 (3.01×10^6^ pfu/ml) (**Figure 3**). The replication efficiency of the GLAF-2 expressing GLV-5b451 strain was similar to that of the parental virus LIVP 6.1.1 in the feline cancer cell line DT09/06 at all tested time points.

### Analysis of anti-VEGF scAb GLAF-2 biosynthesis in GLV-5b451-infected DT09/06 cells

**Figure 4:**
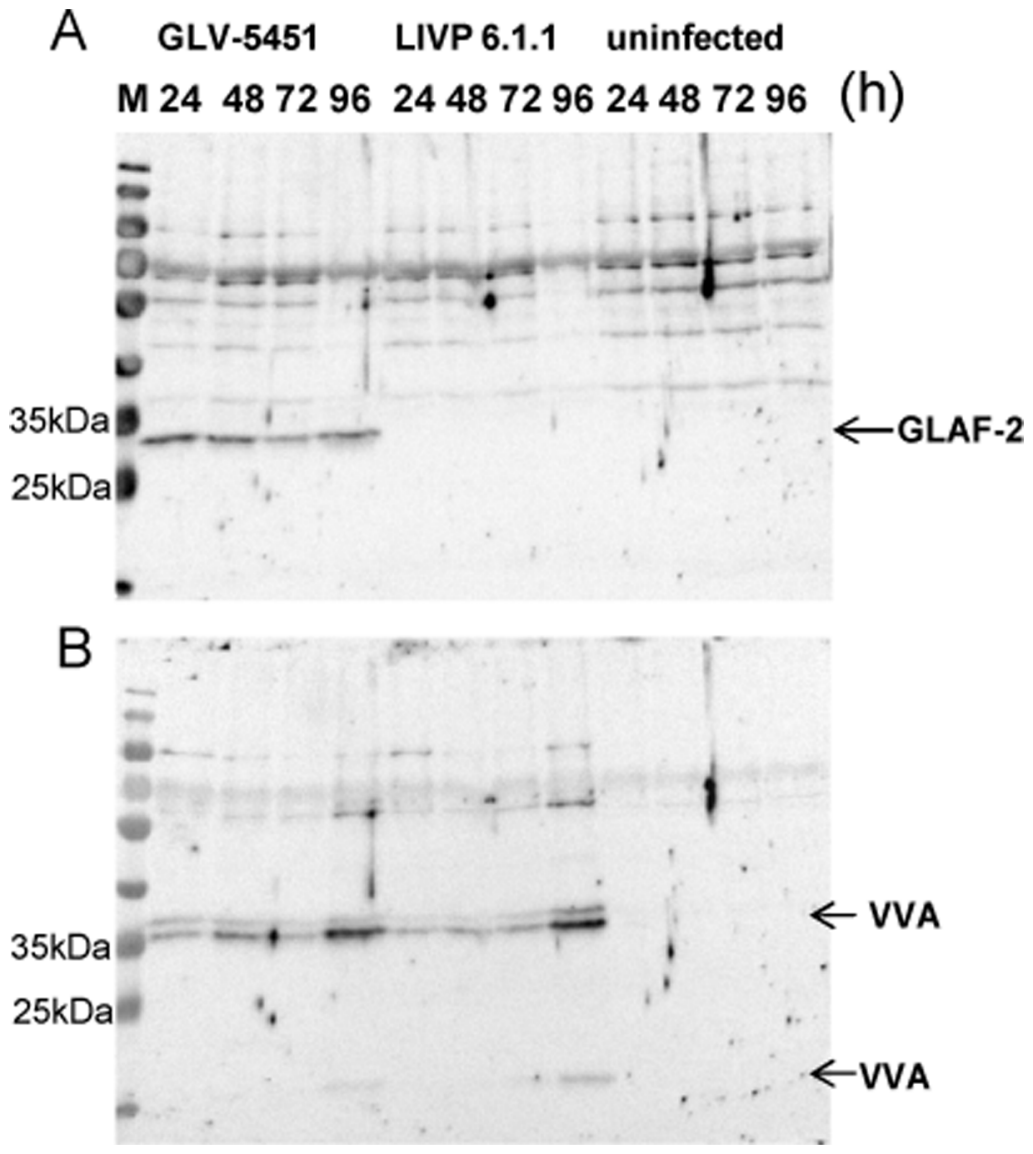
Expression of virus mediated proteins in DT09/06 cells. Western blot analysis of GLV-5b451-, LIVP 6.1.1-infected (MOI of 1.0) or uninfected DT09/06 cells. Protein fractions from cell lysates were isolated at different time points and separated by SDS/PAGE. Western blot analysis was performed as described in material and methods. GLAF-2: anti-VEGF scAb; VVA: Vaccinia virus specific proteins; M: PageRuler Prestained Protein Ladder # 26616 (Thermo Scientific, Bonn, Germany), a mixture of ten blue-, orange-, and green-stained recombinant proteins (10 to 170 kDa), was used as size standards in Western blotting.

DT09/06 cells were infected with GLV-5b451 or LIVP 6.1.1 (control) at an MOI of 1.0 in 24-well plates. At different time points, cells were harvested and analyzed in Western Blot using anti-G6 or anti-vaccinia virus (VV) antibodies, respectively (**Figure 4**). The data clearly demonstrated that GLV-5b451-infected DT09/06 cells expressed the GLAF-2 protein (**Figure 4A**) of expected size (27 kDa). In addition, the GLAF-2 expression correlated well with the expression of vaccinia virus specific proteins (**Figure 4B**). No proteins of similar sizes were detected in uninfected DT09/06 cells (**Figure 4**). This is evidence that the GLAF-2 protein was successfully expressed in infected feline mammary carcinoma DT09/06 cells.

### The GLAF-2 antibody specifically recognizes feline VEGF

**Figure 5:**
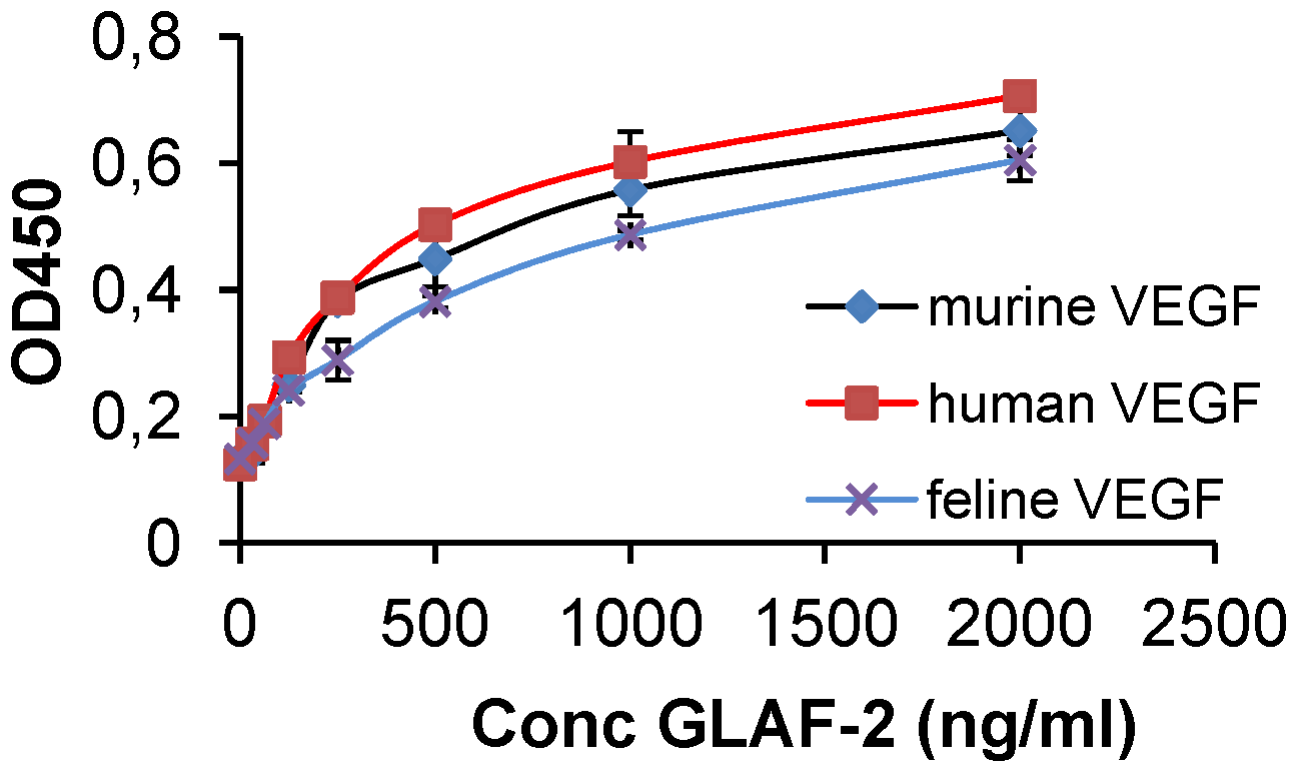
Interactions of purified GLAF-2 antibody with feline, murine, and human VEGFs. Affinity and cross reactivity of GLAF-2 was demonstrated by ELISA. Equal concentrations of feline, murine, or human VEGF (100 ng/well) were coated on ELISA plates. Seven two-fold dilutions of purified GLAF-2 protein ranging from 2000 ng/ml to 31.3 ng/ml were incubated with feline, murine and human VEGFs. PBS was used as negative control. For further ELISA experimental conditions see material and methods. ODs obtained for various conc. of GLAF-2 against feline, murine and human VEGF were plotted. ELISA was repeated in three independent experiments. Each value represents the mean (n = 3) +/− standard deviations (SD).

Since until now the affinity of GLAF-2 to feline (fe) VEGF has not been characterized yet, we tested the ability of GLAF-2 antibody to bind recombinant feline VEGF (R&D System) by ELISA. In these experimental settings we used murine (m) as well as human (h) VEGF as controls. The data demonstrated that this antibody was functional and recognized all tested VEGFs with similar efficiency (**Figure 5**).

### A single systemic administration of GLV-5b451 significantly regresses growth of DT09/06 derived tumors in nude mice

**Figure 6:**
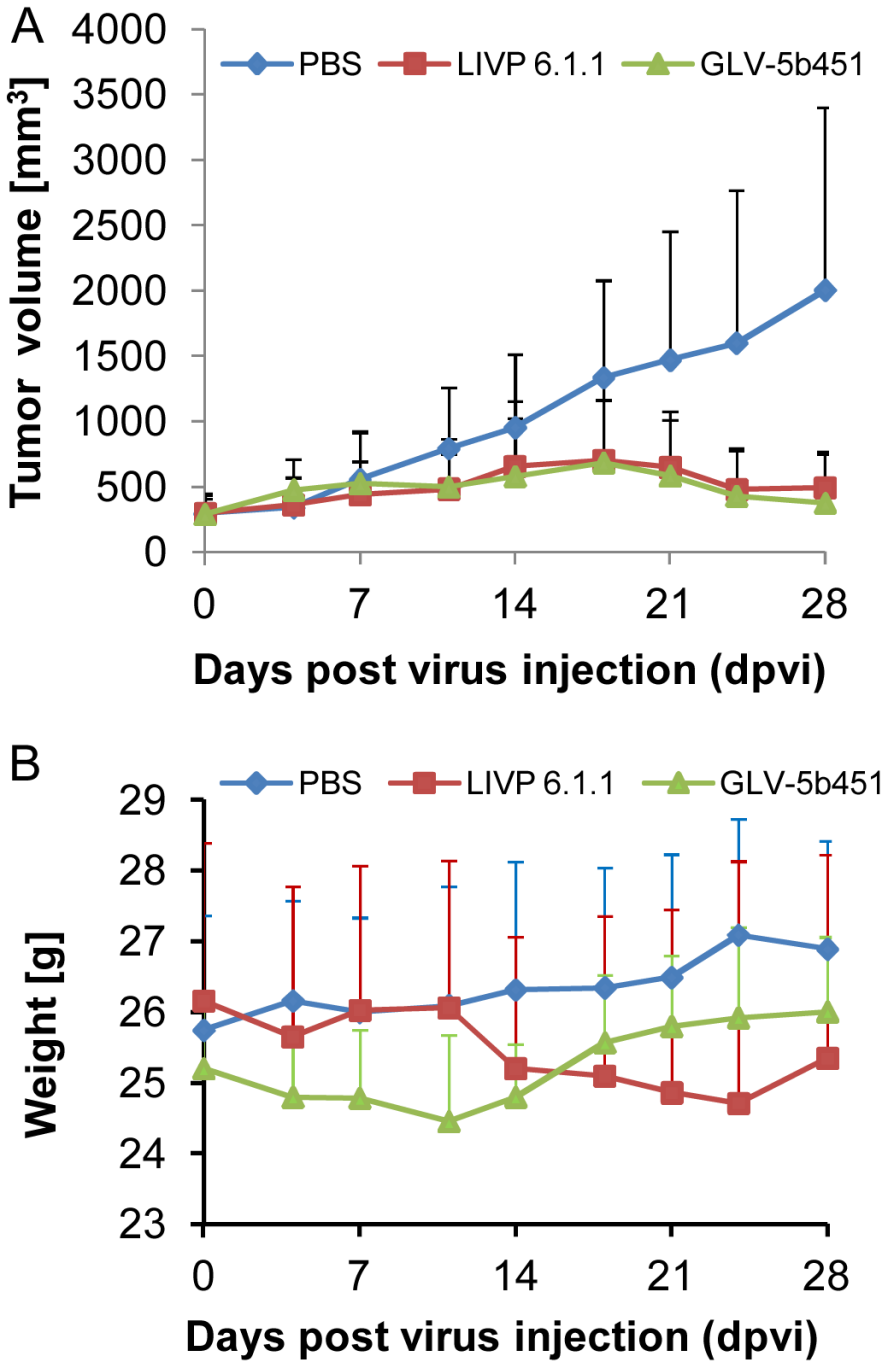
Growth of feline mammary carcinoma tumors in virus- and mock-treated mice. (**A**) Groups of DT09/06 tumor-bearing nude mice (n = 13) were either treated with a single dose of 1×10^7^ pfu GLV-5b451, LIVP 6.1.1. or with PBS (mock control). Tumor size was measured twice a week. Two-way analysis of variance (ANOVA) with Bonferroni post-test was used for comparison of two corresponding data points between groups and presented as mean values +/− SD. (****P<0.0001, **P<0.01). (**B**) Relative mean weight changes of DT09/06 cell xenografted mice after virus or PBS treatment. The data are presented as mean values +/− SD.

Thirty-nine female nude mice at an age of 6–8 weeks were implanted with 1×10^7^ DT09/06 cells. Four weeks post implantation, all mice developed tumors with volumes of 300 to 400 mm^3^. Animals were separated into three groups (n = 13) and were injected with a single dose of GLV-5b451, LIVP 6.1.1 (1×10^7^ pfu in 100 µl PBS) or PBS (100 µl) intravenously (i.v.) into the lateral tail vein. LIVP 6.1.1, a non-GLAF-2 expressing parental virus strain of GLV-5b451 virus, was used as an additional control. Tumor size was measured twice a week. As shown in **Figure 6A**, the virus treatment led to significant differences in tumor growth between PBS controls and all virus-treated mice from 21 to 28 days post virus injection (dpvi). Due to excessive tumor burden, more than 50% of the animals of the PBS control group developed tumors >3000 mm^3^, we terminated the experiment at day 28 post injection. In this experimental setting, we did not find a significant difference between the two virus treated groups (GLV-5b451 vs. LIVP 6.1.1 P = 0.99). In addition, the toxicity of the GLV-5b451 virus was determined by monitoring the relative weight change of mice over time (**Figure 6B**). All virus-treated mice showed stable mean weight over the course of studies. There were no signs of virus-mediated toxicity.

### Biodistribution of GLV-5b451 virus in DT09/06 tumor-bearing nude mice

Three tumor-bearing mice injected with 1×10^7^ pfu of GLV-5b451 were analyzed for virus distribution. Viral titers were determined by standard plaque assays on CV-1 cells using aliquots of the homogenized tissues and were displayed as mean pfu/g organ or tumor tissue (n = 3). **Table 1** summarizes the GLV-5b451 virus distribution in DT09/06 xenografted mice at day 28 post virus injection. The highest viral titers of about 10^7^ pfu/g were identified in primary tumors of virus-treated mice. In contrast, only very few GLV-5b451 virus particles were detected in whole healthy organs (**Table 1**). The data clearly showed that GLV-5b451 virus displayed an enhanced tumor specific replication.

**Table 1:** Biodistribution of GLV-5b451 in virus-treated mice bearing DT09/06 xenografts at 28 days post virus injection (dpvi).

PFU per gram (g) of organ or tumor tissue	DT09/06 xenografts treated with 1×10^7^ pfu GLV-5b451		
**Mouse No**	**288**	**290**	**337**
**Tumor**	4.66E+07	1.35E+07	4.39E+07
**Lung**	1.72E+02	7.6E+02	n.d.
**Liver**	6.94E+03	7.29E+03	2.62E+02
**Spleen**	1.75E+02	8.33E+03	4.33E+02
**Kidney**	1.5E+03	4.71E+02	1.0E+02
**Ovaries**	1.6E+03	n.d.	n.d.

The data were determined by standard plaque assay on CV-1 cells using aliquots of the homogenized organs and were displayed as mean pfu per gram of organ or tissue. For each organ, two aliquots of 0.1 ml were measured in triplicates.

n. d.: not detected (detection limit <10 pfu/organ).

### GLV-5b451 tumor colonization significantly decreases levels of functional VEGF and inhibits development of tumor vasculature

**Figure 7:**
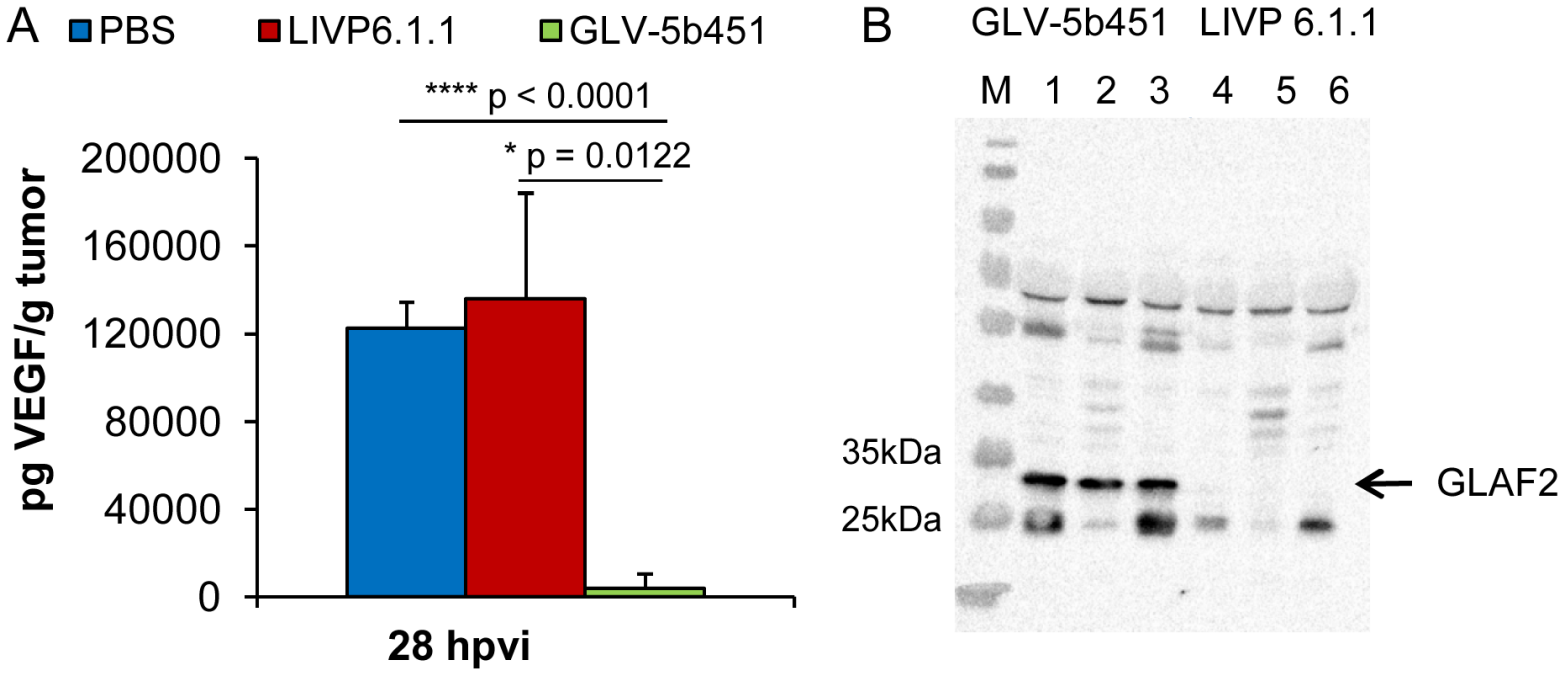
Presence of the scAb GLAF-2 and VEGF in tumors of LIVP 6.1.1- or GLV-5b451- injected DT09/06 xenograft mice. (**A**) Western blot analysis of DT09/06 tumor xenografts injected with LIVP 6.1.1 or GLV-5b451 virus (n = 3). The presence of GLAF-2 proteins was performed as described before. Each sample represents an equivalent of 2 mg tumor mass. (**B**) Levels of functional VEGF in tumor lysates determined by ELISA. The graph was plotted using the mean values of each group of three independent measurements. The data are presented as mean values +/− SD. An unpaired t-test was performed revealing significant differences (****P<0.0001, **P<0.01).

To test the effect of the GLAF-2 antibody expression on tumor angiogenesis, we first analyzed intra-tumoral GLAF-2 and VEGF levels of GLV-5b451- in comparison to LIVP 6.1.1- or PBS-treated DT09/06 tumors. In the presence of GLAF-2 antibodies, we observed a significant decrease of tumoral VEGF concentration in all GLV-5b451-treated mice but not in LIVP 6.1.1- or PBS-injected animals (GLV-5b451 vs. LIVP 6.1.1 *P = 0.0122; GLV-5b451 vs. PBS ****P<0.0001 and PBS vs. LIVP 6.1.1 P = 0.6788) (**Figure 7**).

**Figure 8:**
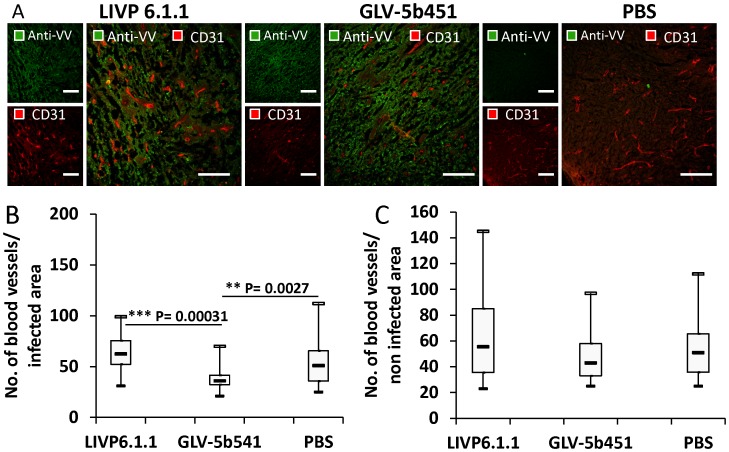
Visualization and determination of vascular density using CD31 immunohistochemistry in virus-treated (LIVP 6.1.1 or GLV-5b451) and PBS-treated tumors at 28 dpvi. (**A**) Tumor sections labeled with anti-CD31 antibody (red) and anti-vaccinia virus (VV) antibody (green). Scale bars: 150 µm. (**B, C**) The vascular density was measured in CD31-labeled tumor cross-sections (n = 3 mice per group, 16 images per mouse) and presented as mean values +/− SD. (***P<0.001, **P<0.01,*P<0.05 Student's t-test).

In addition, tumor angiogenesis was assessed by CD31 immunostaining and microvessel density analysis. For this purpose, CD31-labelled cross sections of tumors from LIVP 6.1.1-, GLV-5b451- and PBS-treated mice were compared by fluorescence microscopy at day 28 after treatment (**Figure 8**). The data revealed that the vascular density of GLV-5b451-infected tumors was significantly decreased in comparison to that of LIVP 6.1.1- and PBS-injected control tumors (GLV-5b451 vs. LIVP 6.1.1 ***P = 0.00031; GLV-5b451 vs. PBS **P = 0.00271) (**Figure 8B**). However, significant reduction of the vascular density was observed in virus-infected areas only (**Figure 8B**; infected) which may serve as evidence that this effect was mediated by the GLAF-2 protein overproduction following virus colonization of tumors. Moreover, the vascular density of infected areas of GLV-5b451 tumors was also significantly lower than that of non-infected areas (infected GLV-5b451 (**Figure 8B**) vs. non infected GLV-5b451 (**Figure 8C**); *P = 0.0484).

The results demonstrate that the virus colonization in combination with scAb GLAF-2 production led to a decrease of tumoral VEGF concentration and local inhibition of the blood vessel development in the GLV-5b451 virus-infected tumor tissue only.

### Effect of virus colonization and tumor vascular density on the presence of immune cells in virus treated DT09/06 tumors

**Figure 9:**
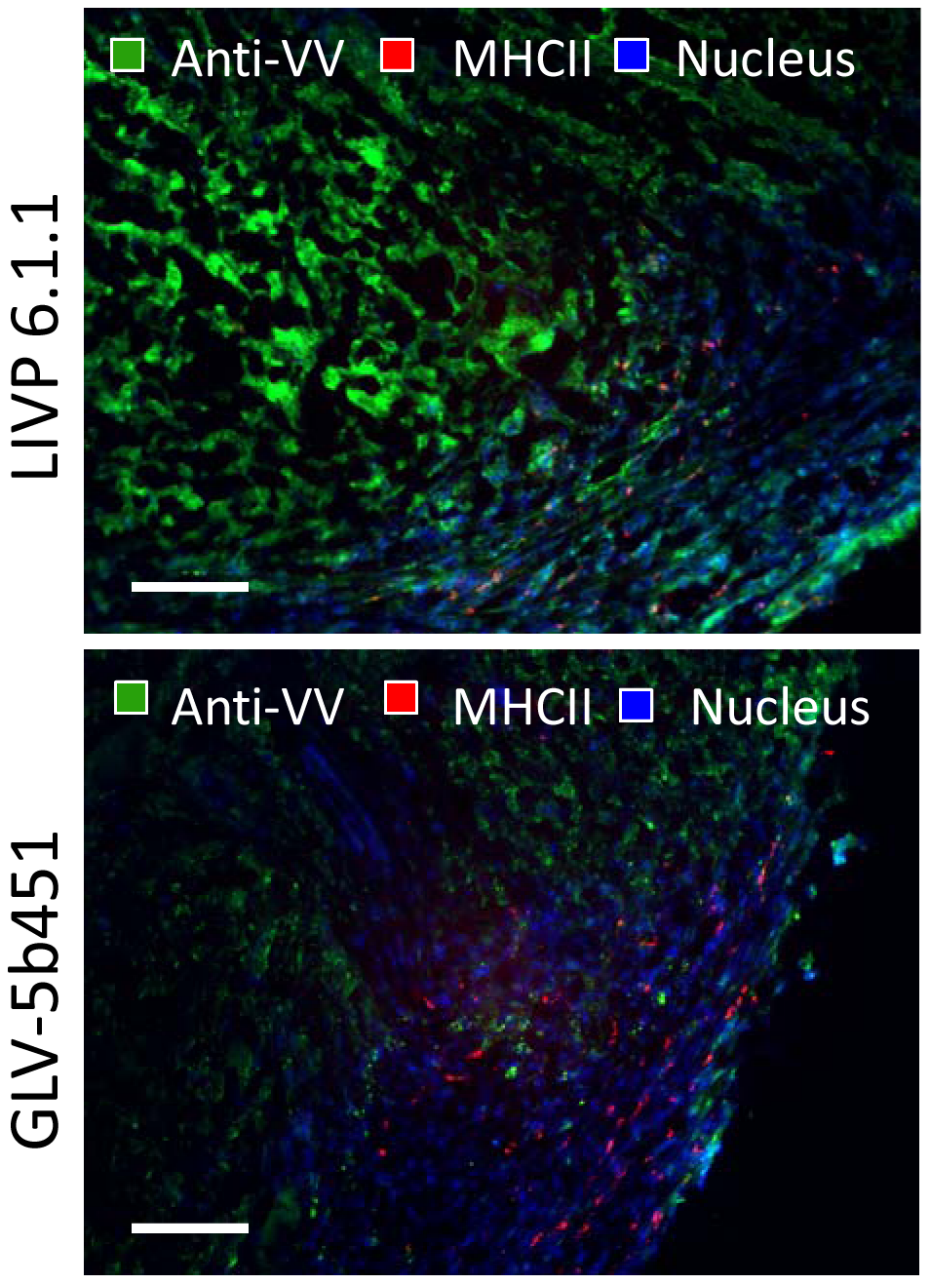
Immunohistochemical staining of either LIVP 6.1.1- or GLV-5b451-treated DT09/06 xenograft tumors at 28 dpvi. Representative tumor sections labeled with anti-vaccinia virus (green), anti-MHCII antibodies (red) and Hoechst 33342 staining (blue). Scale bars: 200 µm.

In the last part of our study, we investigated the effects of the virus infection and tumor vasculature on peri- and intratumoral infiltration of host immune cells in tumors of DT09/06-tumor-bearing mice. Cryosections prepared from DT09/06 tumors resected 28 days after either LIVP 6.1.1 or GLV-5b451 treatment were analyzed for the presence of MHC II-positive host immune cells (B cells, monocytes, macrophages and dendritic cells, **Figure 9**). Interestingly, there was no significant difference in the number of MHC II-positive cells in tumor tissues infected with either LIVP 6.1.1 or GLV-5b451 in the late phase of infection.

In summary, the results suggest that the decreased vascular density after GLV-5b451 treatment did not change significantly the intratumoral infiltration of MHCII-positive immune cells in comparison to the LIVP 6.1.1 treatment.

## Discussion

Despite advances in surgery, radiation and chemotherapy, the available treatment options for mammary carcinoma in cats are limited and the prognosis for patients with advanced-stage disease is very poor. Therefore, developing novel therapies, which may also work synergistically in combination with the conventional treatment options, is crucial. One of the most promising novel cancer agents are oncolytic viruses with therapeutic payloads. Several different oncolytic viruses such as herpes simplex virus, vaccinia virus, seneca valley virus and reovirus are currently in or entering Phase III human clinical trials. In addition, in China the oncolytic adenovirus H101 has been approved for the treatment of human patients with head and neck cancer since 2005 [29]. In contrast, only few such viruses including myxoma virus [30] and poxviruses [8], two distinct members of the family *Poxviridae*, have been tested for feline cancer therapy with moderate success (for a review see [31]).

In the current study, we investigated the oncolytic efficacy of the recombinant vaccinia virus strain GLV-5b451 expressing the anti-VEGF single-chain antibody GLAF-2 in a new feline mammary carcinoma cell line DT09/06 in culture as well as in a novel xenograft model. The results showed that GLV-5b451 was able to effectively infect, replicate in and lyse the DT09/06 cells in culture. In addition, the data revealed that the DT09/06 cell line was tumorigenic in nude mice. About 96% of all implanted mice developed tumors at the site of injection. However, none of the tumor-bearing mice showed any signs of metastasis or of invasive growth pathologically. We therefore assumed that in this case, time for metastasis formation exceeded the time limitation owed to local tumor growth in mice. Interestingly, the feline donor had evidence of lymphatic metastases. Taken together, this xenograft model may become an useful tool for preclinical studies for treatment of FMC.

We were able to demonstrate that treatment with the oncolytic vaccinia virus GLV-5b451 harbouring the gene for the anti-VEGF scAb GLAF-2 significantly reduced the growth of feline mammary carcinoma xenografts predominantly by oncolysis and inhibited tumor angiogenesis simultaneously. VEGF is an important regulator of tumor angiogenesis and its pathway has been targeted with antibodies and small molecules [12], [13]. One of the best characterized strategies is the VEGF blockade using the humanized anti-VEGF monoclonal antibody (mAb) bevacizumab (avastin) [32]. However, despite very promising preclinical results, bevacizumab has not been shown to provide a benefit in patients with breast cancer as monotherapy (http://www.fda.gov/NewsEvents/Newsroom/PressAnnouncements/ucm279485.htm).

Another new strategy is to fight tumor vasculature with oncolytic viruses. This method has already been successfully used in several different cancer types (for a recent review see [33]). In the current study, we utilized the combination of oncolytic vaccinia virus and single chain antibody targeting VEGF in order to improve the anti-angiogenic and vascular-disrupting properties of our oncolytic virus. We have recently reported that treatments with recombinant vaccinia virus strains (VACV) expressing anti-VEGF antibodies (GLAF-1 or GLAF-2) led to enhanced tumor growth inhibition and vascular disruption in different xenograft models [10], [11], [18]. GLAF-1 and GLAF-2 are identical with the only difference that GLAF-1 contains a FLAG-tag for purification purposes [11]. Here, using Western blot analysis and VEGF ELISA we documented that the presence of the anti-VEGF scAb led to significant decrease of tumoral VEGF protein concentration in GLV-5b451 virus-colonized tumors only (**Figure 7**). The significant higher VEGF concentrations in LIVP 6.1.1-virus-infected tumors confirmed the assumption that presence of GLAF-2 in the tumor tissue alone leads to the reduction of local VEGF level. This fact is also an evidence for the functionality of the GLAF-2 antibody in tumor tissue. We selected DT09/06 xenografted mice to study the effects of the GLAF-2 scAb on tumor angiogenesis since the feline mammary carcinoma DT09/06 cell line showed a constitutively high VEGF expression under cell culture conditions (**Figure 2**).

To be sure that the GLAF-2 antibody is functional in feline mammary carcinoma xenografts, we tested the effect of GLAF-2 on tumor angiogenesis by CD31 immunohistological staining of DT09/06 tumor sections. CD31 is used primarily to demonstrate the presence of endothelial cells in tumor tissue sections and is an effective tool for analysis of microvessel density. The data revealed a significant decrease in the number of blood vessels in GLV-5b451 infected tumors when compared to LIVP 6.1.1- and PBS-injected control tumors at 28 dpvi (**Figure 8**). In the case of LIVP 6.1.1, the non GLAF-2-expressing parental virus of GLV-5b451, no reduction of the vascular density was observed. Interestingly, the significant reduction in vascular density in GLV-5b451 colonized tumors was observed only in virus-infected areas of the tumors. The reason for this localized effect could be the reduced number of microvessels in DT09/06 tumors that might affect the intratumoral spread of GLAF-2 protein. Very similar data were obtained with GLAF-1 antibody in canine STSA-1 xenograft model [18].

We have also shown for the first time that GLAF-2 can specifically bind to feline VEGF (**Figure 5**). In addition, the cross reactivity of GLAF-2 with murine VEGF was demonstrated. The GLAF-2-binding to VEGF from both feline and mouse origin is advantageous in our feline DT09/06 xenograft model, as blocking of VEGF from both species could be important to enhance therapeutic efficacy [29].

In the last part of our study we investigated the effect of virus colonization and the tumoral vascular density on the peri- and intratumoral infiltration of MHC II-positive host immune cells. Interestingly, despite a significant reduction of vascular density in GLV-5b451-treated tumors, we did not notice a significant difference in the number of MHC II-positive cells in comparison to LIVP 6.1.1-injected control tumors (**Figure 9**). These findings suggest that the reduced vascular density in GLV-5b451-treated xenografts is not crucial for intratumoral infiltration of host immune cells at least in the late phase of infection. The presence of host immune cells (like e.g. macrophages and dendritic cells) surrounding virus-infected cancer cells could serve as an evidence for a possible association between vaccinia virus colonization, activation of the host innate immune system and xenograft eradication. Moreover, we and others have recently reported that these interactions may increase the activation and strength of host antitumor immune responses [25], [34]–[37].

Thus, the anti-tumor mechanism in DT09/06 xenografts could be a combination of the direct viral oncolysis of tumor cells and the virus-dependent infiltration of tumor-associated host immune cells. The observed significant reduction in vascular density in GLV-5b451 colonized tumors compared to LIVP 6.1.1. tumors seems to be not essential for the tumor growth inhibition at last till 28 dpvi, since we did not find a significant difference between the both virus treated groups (GLV-5b451 vs. LIVP 6.1.1 P = 0.99). However, the inhibition of angiogenesis could be an important anti-tumoral mechanism in immunocompetent patients.

In conclusion, oncolytic vaccinia virus strains and especially GLV-5b451 may be promising candidates for therapy of feline cancer patients with diagnosed mammary carcinoma.

## Supporting Information

Figure S1 **Analysis of feline mammary carcinoma cells or tumor tissue by transmitted light microscopy (A), doubling time of DT09/06 cells in cell culture (B), histology (C) or PCR (D).** (**A**) Transmitted light microscopy of uninfected feline mammary carcinoma DT09/06 cells in MEM-C culture (×100 magnification). (**B**) Cell counts used to determine population doubling time of DT09/06 cells. Cells were seeded in 12-well plates with a seeding concentration of 1×10^4^ or 2×10^4^ DT09/06 cells per ml in triplicates (n = 3). The cells were harvested after 24, 48, 72 and 96 hours, respectively, and the mean cell numbers and standard deviations were determined. The points were plotted using the 24 to 96 h time points. The exponential trend lines were drawn and the coefficients of determination (R^2^) specified. The population doubling time was identified using the calculator found on www.doublingtime.com/compute.php. The identified population doubling times were 21.50 h (seeding density of 1×10^4^/well) and 23.43 h (seeding density of 2×10^4^/well). The doubling time was 22.46 h under these cell culture condition. (**C**) Histological section of a DT09/06 xenograft, right flank, athymic nude mouse (H&E, ×200-magnification). (**D**) Electrophoretic analysis of the 12S rRNA PCR products on 1.6% agarose gels containing Midori Green (Nippon Genetics Europe GmbH, Düren, Germany). Identification of cat and mouse tissues by Duplex PCR with primers either for feline 12S rRNA gene (**F**; forward: 5′-AATTGAATCGGGCCATGAA-3′ and reverse: 5′- CGACTTATCTCCTCTTGTGGGTGT-3′), or for murine 12S rRNA gene (**M**; forward: 5′-AAATCCAACTTATATGTGAAAATTCATTGT-3′ and reverse: 5′- TGGGTCTTTAGCTATCGTCGATCAT-3′). The primers designed generated specific fragments of 108 or 96 bp in length for cat or mouse tissues, respectively [23]. Lanes: **1**: PCR Marker (BioLabs); **2**: DT09/06 tumor/**F**-primers; **3**: DT09/06 tumor/M- primers, **4**: DT09/06 cells/F-primers; **5**: DT09/06 cells/M-primer. Molecular sizes are indicated.
(TIF)

Figure S2 **(A) Viability of feline lymphoma F1B cells after LIVP 6.1.1 or GLV-5b451 infection.** 1×10^4^ F1B cells were seeded in 96-well plates and infected with LIVP 6.1.1 and GLV-5b451 at MOI of 1.0. The amount of viable cells was measured using 2,3-bis[2-methoxy-4-nitro-5-sulfophenyl]-2H-tetrazolium-5-carboxanilide inner salt (XTT) assay (Cell Proliferation Kit II, Roche Diagnostics, Mannheim, Germany), according to the manufacturer's protocol at different time points after infection. Quantification of cell viability was performed in an ELISA plate reader (SpectraMax M5, Molecular Devices, Sunnyvale, USA) at 450 nm with a reference wavelength of 700 nm. Viral cytotoxicity was measured at Day 0, 1, 3, 5, 7 and 9. Mean values (n = 4) and standard deviations are presented as percentages of the respective uninfected controls defined as 100% viable. **(B) Viability of canine mammary MTH52c carcinoma cells after LIVP 6.1.1 or GLV-5b451 infection at MOIs of 0.1 and 1.0, respectively.** 4×10^5^ MTH52c cells were seeded in 24-well plates and infected with LIVP 6.1.1 and GLV-5b451 at MOIs of 0.1 and 1, respectively. The fraction of viable cells after 24, 48, 72 and 96 hours was detected using 3-(4, 5-dimethylthiazol-2-yl)-2, 5-diphenyltetrazolium-bromide (MTT). Mean values (n = 3) and standard deviations are presented as percentages of the respective uninfected controls defined as 100% viable. The data represent two independent experiments. There were no significant differences between groups (P>0.05).
(TIF)
