# Supplementary figures and images for: Evaluation of a New Recombinant Oncolytic Vaccinia Virus Strain GLV-5b451 for Feline Mammary Carcinoma Therapy
Source: PLoS One. 2014 Aug 5;9(8):e104337. doi: 10.1371/journal.pone.0104337 (PMC4122492; doi:10.1371/journal.pone.0104337)

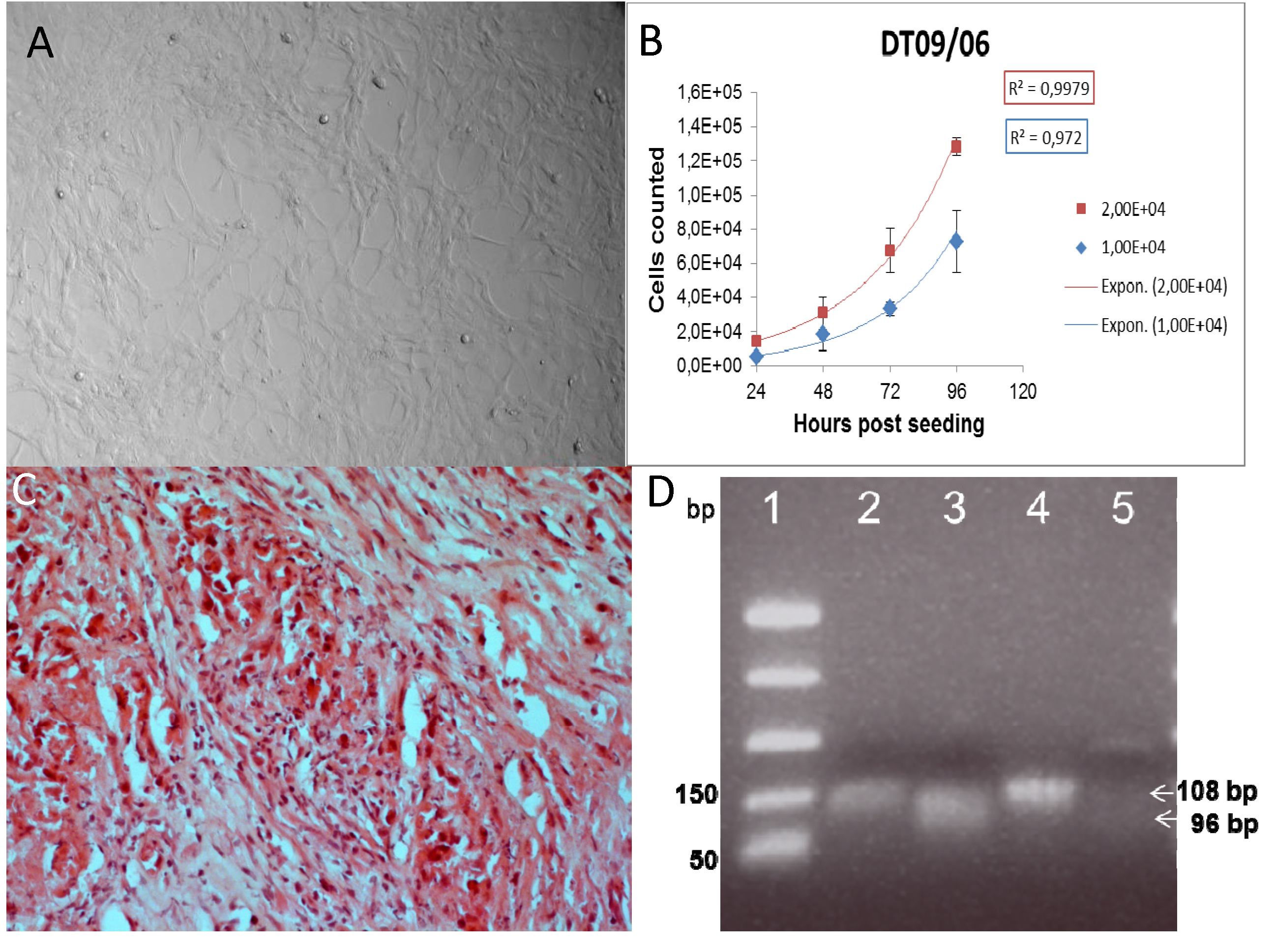

Supplement: Figure S1 — Analysis of feline mammary carcinoma cells or tumor tissue by transmitted light microscopy (A), doubling time of DT09/06 cells in cell culture (B), histology (C) or PCR (D). (A) Transmitted light microscopy of uninfected feline mammary carcinoma DT09/06 cells in MEM-C culture (×100 magnification). (B) Cell counts used to determine population doubling time of DT09/06 cells. Cells were seeded in 12-well plates with a seeding concentration of 1×10^4^ or 2×10^4^ DT09/06 cells per ml in triplicates (n = 3). The cells were harvested after 24, 48, 72 and 96 hours, respectively, and the mean cell numbers and standard deviations were determined. The points were plotted using the 24 to 96 h time points. The exponential trend lines were drawn and the coefficients of determination (R2) specified. The population doubling time was identified using the calculator found on www.doublingtime.com/compute.php. The identified population doubling times were 21.50 h (seeding density of 1×10^4^/well) and 23.43 h (seeding density of 2×10^4^/well). The doubling time was 22.46 h under these cell culture condition. (C) Histological section of a DT09/06 xenograft, right flank, athymic nude mouse (H&E, ×200-magnification). (D) Electrophoretic analysis of the 12S rRNA PCR products on 1.6% agarose gels containing Midori Green (Nippon Genetics Europe GmbH, Düren, Germany). Identification of cat and mouse tissues by Duplex PCR with primers either for feline 12S rRNA gene (F; forward: 5′-AATTGAATCGGGCCATGAA-3′ and reverse: 5′- CGACTTATCTCCTCTTGTGGGTGT-3′), or for murine 12S rRNA gene (M; forward: 5′-AAATCCAACTTATATGTGAAAATTCATTGT-3′ and reverse: 5′- TGGGTCTTTAGCTATCGTCGATCAT-3′). The primers designed generated specific fragments of 108 or 96 bp in length for cat or mouse tissues, respectively [23]. Lanes: 1: PCR Marker (BioLabs); 2: DT09/06 tumor/F-primers; 3: DT09/06 tumor/M- primers, 4: DT09/06 cells/F-primers; 5: DT09/06 cells/M-primer. Molecular sizes are indicated. (TIF) [file pone.0104337.s001.tif]

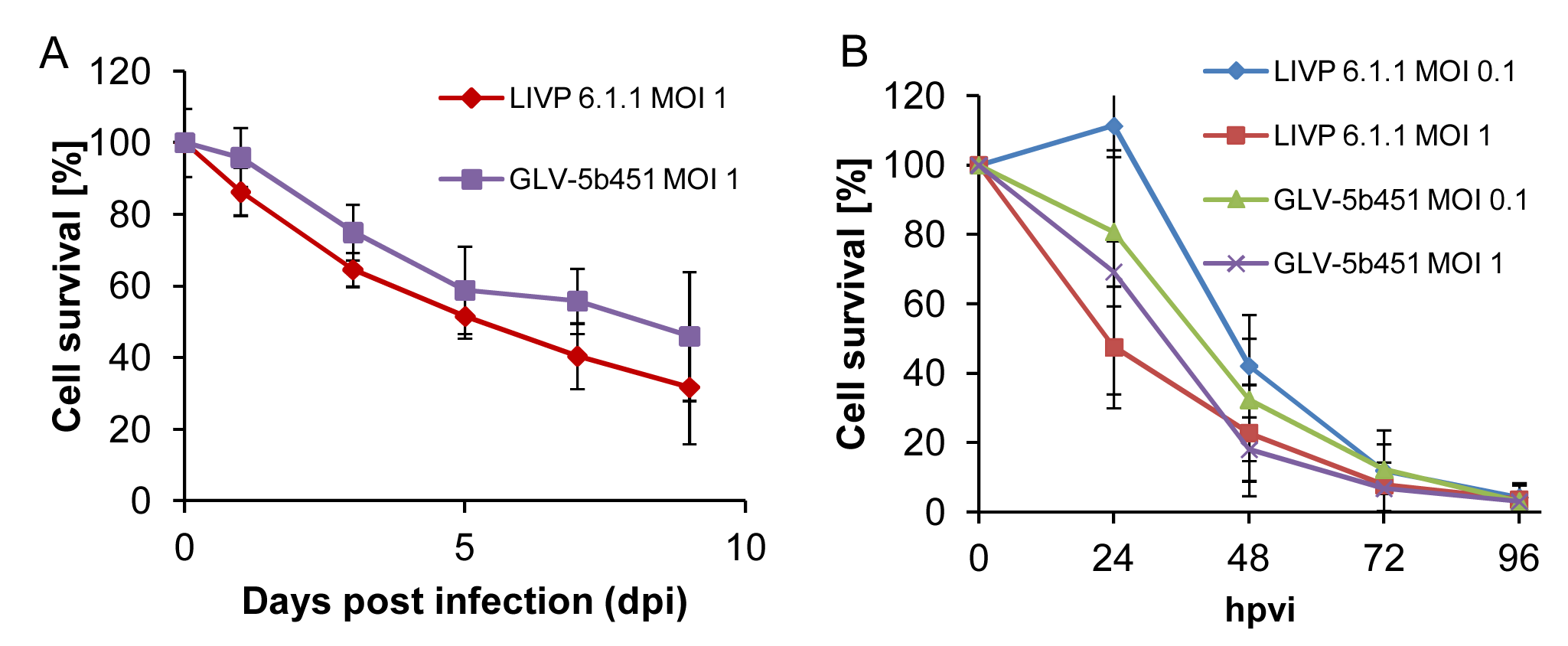

Supplement: Figure S2 — (A) Viability of feline lymphoma F1B cells after LIVP 6.1.1 or GLV-5b451 infection. 1×10^4^ F1B cells were seeded in 96-well plates and infected with LIVP 6.1.1 and GLV-5b451 at MOI of 1.0. The amount of viable cells was measured using 2,3-bis[2-methoxy-4-nitro-5-sulfophenyl]-2H-tetrazolium-5-carboxanilide inner salt (XTT) assay (Cell Proliferation Kit II, Roche Diagnostics, Mannheim, Germany), according to the manufacturer's protocol at different time points after infection. Quantification of cell viability was performed in an ELISA plate reader (SpectraMax M5, Molecular Devices, Sunnyvale, USA) at 450 nm with a reference wavelength of 700 nm. Viral cytotoxicity was measured at Day 0, 1, 3, 5, 7 and 9. Mean values (n = 4) and standard deviations are presented as percentages of the respective uninfected controls defined as 100% viable. (B) Viability of canine mammary MTH52c carcinoma cells after LIVP 6.1.1 or GLV-5b451 infection at MOIs of 0.1 and 1.0, respectively. 4×10^5^ MTH52c cells were seeded in 24-well plates and infected with LIVP 6.1.1 and GLV-5b451 at MOIs of 0.1 and 1, respectively. The fraction of viable cells after 24, 48, 72 and 96 hours was detected using 3-(4, 5-dimethylthiazol-2-yl)-2, 5-diphenyltetrazolium-bromide (MTT). Mean values (n = 3) and standard deviations are presented as percentages of the respective uninfected controls defined as 100% viable. The data represent two independent experiments. There were no significant differences between groups (P>0.05). (TIF) [file pone.0104337.s002.tif]
